# Social Participation and Mental Health in the Establishment Programme for Newly Arrived Refugees in Sweden—A Document Analysis

**DOI:** 10.3390/ijerph19084518

**Published:** 2022-04-08

**Authors:** Sofia Larsson, David Gunnarsson, Linda Vikdahl

**Affiliations:** 1 Department of Health Sciences, The Swedish Red Cross University College, Hälsovägen 11, SE-141 57 Huddinge, Sweden; david.gunnarsson@sh.se (D.G.); linda.vikdahl@sh.se (L.V.); 2 School of Historical and Contemporary Studies, Södertörn University, Alfred Nobels Allé 7, SE-141 89 Huddinge, Sweden

**Keywords:** mental health, social participation, labour market, newly arrived refugees, the Swedish Public Employment Service, establishment programme

## Abstract

Newly arrived refugees and asylum seekers constitute a vulnerable population in terms of health and social conditions due to lived trauma and experiences of loss, as well as factors in the host country such as not speaking the language, not having employment and social exclusion. Studies have shown that many newly arrived refugees find it difficult to establish a sustainable position in the host country’s labour market due to a lack of connections, low levels of education and political, social and cultural barriers. The Swedish Public Employment Service runs an establishment programme aimed at helping newly arrived refugees to find employment quickly and manage their own livelihoods. In this study, we analyse the administrator support document used by Swedish Public Employment Service case workers in their work with the programme to explore whether and how it considers the participants’ mental health and conditions for social participation. The results show that despite newly arrived refugees being especially vulnerable in terms of mental health, little attention is paid to these aspects, the possible effects they may have on the programme, the participants’ integration into the labour market and Swedish society as a whole.

## 1. Introduction

Refugees and asylum seekers constitute a particularly vulnerable group of migrants in terms of health and social conditions. For instance, studies have shown that refugees resettled in Western countries are much more likely to experience depression, anxiety and/or post-traumatic stress disorders than age-matched general populations in those countries [1,2]

In the existing research literature, studies of factors in the host country that affect the mental health and socio-economic integration of refugees are scarce [3]. However, it is clear that not being able to speak the host society’s language, not having a job and being socially excluded increase the risk of mental ill-health and distress [4]. Likewise, social participation, such as community involvement and participation in various social activities, networks and communities after migration, is important for increasing well-being and reducing mental illness [3].

In Sweden, the Public Employment Service runs an establishment programme that provides activities and training for newly arrived refugees in Sweden to help them to learn Swedish quickly, find employment and manage their own livelihoods. By analysing the administrator support document, which is the principal guiding document used by case workers working with participants in the establishment programme, the study described in this article explores whether, how and the extent to which the programme takes the participants’ mental health and conditions for social participation into account.

## 2. Background

### 2.1. Refugees and Asylum in Sweden

In accordance with the UN Convention and Protocol Relating to the Status of Refugees, EU regulations and Swedish legislation, a person is considered to be a refugee if they have crossed an international border to find safety in another country due to war, violence, conflict and/or persecution because of race, nationality, religious or political beliefs, gender, sexual orientation or affiliation to a particular social group [5]. 

In the early 2000s, some 30,000 people applied for asylum in Sweden each year. In 2012, these numbers started to increase, and in 2015, the number of asylum seekers was estimated to be at an all-time high of over 160,000, with many fleeing from the war in Syria. Since 2015, the possibility of entering Sweden has been reduced due to political decisions and, as a result, the number of asylum seekers has decreased. In 2020, around 13,000 people sought asylum in Sweden, and by 2021, Afghanistan topped the list of the most common citizenship countries among asylum seekers, followed by Syria and Iraq. Of all asylum seekers in Sweden, men make up about 60 per cent [6]. Immigrant groups with a large proportion of only pre-secondary education have largely come to Sweden for refugee reasons. Among people from Somalia and Afghanistan, just over 60 per cent have pre-secondary education as their highest level of education, and this is about 50 per cent among people from Eritrea. Of immigrants from Syria and Iraq, about 40 percent have pre-secondary education at most, but about the same number have post-secondary education [7].

In Sweden, all asylum applications are assessed individually, and a person who meets all the refugee criteria is granted a refugee status declaration, which is an internationally recognised status under the UN Refugee Convention. According to the Temporary Act that was part of a governmental policy package announced in 2015 and valid until 19 July 2021, a person with a refugee status declaration was normally given a 3-year residence permit in Sweden. This could be extended if the person met one or several of the set criteria, such as being in need of protection or being financially able to support oneself as an employee or as a self-employed individual in Sweden [5,8,9]. The Temporary Act was introduced after the refugee situation in 2015 to reduce the number of asylum seekers in Sweden. In short, the Temporary Act included fewer grounds for protection, temporary residence permits as a general rule rather than permanent ones, limited opportunities for family reunification and stricter maintenance requirements for family reunification [10].

For those who have recently been granted refugee status in Sweden, a residence permit and have been received in a Swedish municipality, the term newly arrived is used by government agencies. Newly arrived refugees are covered by an Establishment Initiatives Act for a period of two years, which entitles them to professional support for learning Swedish, finding employment and managing their own livelihoods [11].

### 2.2. Mental Health among Newly Arrived Refugees

The World Health Organization (WHO) defines health as “a state of complete physical, mental and social well-being and not merely the absence of disease or infirmity” and further states that definitions of mental health include “subjective well-being, perceived self-efficacy, autonomy, competence, intergenerational dependence and recognition of the ability to realize one’s intellectual and emotional potential”. WHO also recognises that mental health can be defined as “a state of well-being whereby individuals recognize their abilities, are able to cope with the normal stresses of life, work productively and fruitfully, and contribute to their communities” [12]. Furthermore, public health is defined as “the art and science of preventing disease, prolonging life and promoting health through the organized efforts of society” [12].

Refugees and asylum seekers constitute a particularly vulnerable group of the migrant population in terms of health and social conditions. In comparison with other migrants, the migration process for refugees is characterised by difficult events, challenges and sources of serious psychological strain and stress before, during and after migration. For instance, a study from 2005 shows that refugees resettled in Western countries were ten times more likely to suffer from post-traumatic stress disorders than age-matched general populations in those countries [1]. Furthermore, a relatively recent Swedish survey of newly arrived refugees from Syria shows that approximately one third suffer from severe depression, anxiety and/or post-traumatic stress disorder (PTSD) [2]. 

Several international research studies of refugees’ health cover mental health [13], the severe traumas that form the background to flight and forced migration and how this could affect refugees’ lives in the new country [1,13,14,15]. For example, it has been shown that mental illness due to refugee trauma can affect people’s sleep patterns and cognitive functions, thereby making it more difficult to learn a new language, complete an education or stay in employment [16].

In comparison, efforts to understand the factors in the host country that affect the mental health and socio-economic integration of refugees are less prominent in the research literature [3]. However, work is a major determinant of mental health and a socially integrating force [17], and long-term unemployment can increase the risk of ill health and especially mental illness [18], while a lack of social participation has been shown to contribute to psychosocial stress due to isolation and exclusion [19]. Furthermore, the psychological consequences of unemployment include impaired psychological well-being, anxiety, depression, reduced self-confidence, social isolation and reduced levels of activity [20]. 

Despite strong workers’ rights in Sweden, such as the right to at least 25 days of paid vacation per year, 480 days of paid paternity leave per child, as well as paid sick leave [21], mental illness in the form of depression, anxiety disorders and fatigue syndrome are common in the working population. Since 2014, psychiatric diagnoses have been the most common diagnostic group among Swedish workers receiving sick leave [22].

The same report states that factors such as working in precarious employment, working in environments with few opportunities to influence and with a lack of human support, in combination with high requirements and little reward, as well as working in environments with conflict, have negative impact on mental health [22]. In addition to these risk factors that apply to the general working population, newly arrived refugees may experience exclusion on the labour market, and in Swedish society in general, due to not being able to speak the host country’s language, discrimination and due to having little or no access to important social resources, all of which imply an increased risk of mental ill-health [4].

Key domains, including, for instance, employment and legal status, have been used to understand the process and/or the outcome of refugees’ relations to the new social environments of the host society [3,23,24]. It has also been shown that community involvement and participation in various social activities, networks and communities after migration is important for increasing well-being and reducing mental illness among refugees [25,26]. Furthermore, the concept of social participation has been linked to the formation of social capital [27,28], i.e., the collective resources that individuals have access to through their relationships and contacts with others in social networks and that have beneficial effects on their health. For instance, engagement in communities consisting of individuals with the same ethnic background has been shown to improve integration and access to the host country’s labour market [3].

### 2.3. Employment among Refugees

In addition to the personal and societal gains related to employment, whether or not an adult refugee is perceived as having successfully integrated into the receiving country’s society is often dependent on whether they have secured employment [29]. However, refugees tend to encounter difficulties when trying to establish a sustainable position in the host country’s labour market [30,31]. On average, refugees have lower employment rates, more unstable employment contracts and lower incomes than native Swedes. In particular, refugee women, the young and those arriving late in their working lives experience such problems due to economic, political, social and cultural barriers [32].

In 2018, the proportion of unemployed migrants aged 20–64 years in Sweden was 15 per cent, which was about 12 per cent higher than that for native Swedes (3 per cent). The employment rate for migrant women aged 20–64 years was 15.3 per cent. The comparable native women’s unemployment rate at the same age was 2.8 per cent [33,34].

The level of education also affects a person’s chance of finding employment. Men and women aged 15–74 years with no secondary education have an unemployment rate of less than 34 per cent [33,34]. Newly arrived refugees who have been granted asylum in Sweden have a significantly lower employment rate than many other groups [35]. People who migrate to Sweden as refugees rarely have any connection to the labour market. According to Statistics Sweden, most refugees are engaged in education or integration programmes during their first couple of years in Sweden, such as language training classes, and after eight years in the country, about half of all refugees have found employment. Of these, the employment rate is highest for men with at least upper secondary education and lowest among women with only pre-upper secondary education [36].

### 2.4. The Swedish Establishment Programme

In 2010, as part of the governmental establishment reform, the Swedish state took over responsibility for the establishment of newly arrived refugees from the municipalities (Act 2010:197 on establishment initiatives for certain newly arrived immigrants). This reform meant that from then on, the Swedish Public Employment Service had coordination responsibility for the measures aimed at accelerating newly arrived refugees’ establishment in working and social life in Sweden. This included guiding the participants and ensuring that they all had an individual establishment plan with suitable activities, such as educational and participatory measures, and to follow up on these efforts. The assignment also included supporting and helping the organisers of the offered activities (such as municipalities). Important prerequisites for successful establishment are good coordination and individual adaptation, and positive effects were achieved by means of increased case worker density and intensified job search assistance and coaching [37]. 

In January 2018, a new set of rules for the Swedish Public Employment Service came into force, which led to the creation of the current establishment programme (based on Act 2017: 584 on the responsibility for establishment initiatives for newly arrived migrants, Ordinance 2017: 820 on establishment initiatives for newly arrived migrants and Ordinance 2017: 819 on compensation for participants in labour market policy initiatives). The changes that were implemented in 2018 aimed to increase newly arrived individuals’ responsibility for establishing themselves in the labour market by making the regulations more in line with those applicable to other groups of jobseekers. Furthermore, the new regulations were introduced to reduce the administrative workload of case workers, for instance, by moving the handling of the establishment allowance from the Swedish Public Employment Service to the Swedish Social Insurance Agency [38]. The changes also gave administrators more opportunity than before to decide on sanctions in the form of reduced or withdrawn compensation if the participants did not follow the programme as agreed [30,35]. Another prominent feature in the programme is the education requirement, meaning that if a participant does not have any formal schooling, has only attended primary school, or has not studied at the same level as the Swedish upper secondary school, they may have to undertake compulsory education at an adult education institution [39].

In sum, the current establishment programme is aimed at individuals between the ages of 20 and 64 years who have been granted a residence permit, and offers support in the form of language training, social orientation, a structured assessment and evaluation of already acquired knowledge and competencies, access to relevant courses and job training and help with active job searches and guidance if they are considering starting a business [39]. The programme is normally full-time for a period of up to two years [30], but if a participant is on part-time parental leave, works part-time, or is unable to work full-time due to illness or disability, they can take part in the programme on a part-time basis. When enrolled in the establishment programme, participants are entitled to financial support from the Swedish Social Insurance Agency [39].

Statistics show that up to and including the first half of 2017, the number of participants in the establishment initiatives increased. The highest level was reached in July 2017, with just over 73,700 participants. In December 2017, the numbers began to decrease, and by the end of 2019, around 33,000 people were enrolled in the programme. This change in the number of participants happened at approximately the same time as the new establishment programme was introduced in 2018. It would appear that the reduction in participants was primarily due to a reduced inflow into the programme, which in turn can be linked to the fact that the number of asylum seekers who were entitled to a residence permit as refugees or as relatives of refugees had decreased [40]. 

The data relating to the relationship between participation in various activities and the probability of being employed show that subsidised employment and participation in adult education are positively correlated with the probability of being employed in the fourth year after municipal placement. However, women do not participate to the same extent as men, and on average seem to spend more time in the establishment programme, mainly due to parental leave, which could explain their slower pace of establishment in the labour market. A report by the Expert Group on Public Economics (ESO) also shows that women with four or more children have little financial incentive to work due to the design of the so-called establishment supplements [41]. To be granted an extended residence permit, newly arrived refugees need to prove that they can support themselves and their families as employees or as self-employed individuals in Sweden. In many families, it is the men who take responsibility as the breadwinner, as they are perceived as having a greater chance of succeeding, while the women tend to take care of the children and household [42]. With the introduction of the establishment programme in 2018, one of the aims was to counteract this difference in participation and access to the labour market between women and men [43].

## 3. Aim

When defining health as not only the absence of disease but also as an interplay between the individual and society, where societal arenas provide resources for individuals to live their lives as healthily as possible, it is vital to take the role of laws and institutions into account [3]. There also needs to be an awareness that different groups may be affected differently by or have uneven access to them. As stated earlier in the article, previous research has shown that newly arrived refugees are a particularly vulnerable group, and it is therefore possible that changes in the laws and central documents relating to governmental establishment efforts aimed at this group may affect the mental health of its participants.

Hence, the aim of this study was to explore whether and how the administrator support document that is currently used by case workers in the Swedish establishment programme takes the target group’s specific circumstances and conditions for social participation and mental health into account. In doing so, we also made comparisons with the equivalent documents used by the agency’s staff prior to the changes implemented in 2018 in order to determine whether these changes have had any bearing on how the mental health of the participants is approached.

## 4. Material

The main document analysed in this study was the administrator support document used by the Swedish Public Employment Service case workers in their work with newly arrived refugees taking part in the establishment programme [43]. This document was retrieved after a formal request to the authority to provide the research team (the authors of this paper) with the policy and strategy documents relevant to its work, promoting the establishment of newly arrived refugees in the Swedish labour market. The Swedish Public Employment Service’s administrator support document runs to sixty-seven pages and describes the establishment programme’s purpose, the target group and the activities that are offered. The document also functions as a step-by-step manual to help the agency’s case workers to guide participants through the establishment programme. It also gives bureaucratic and technical directives, such as which questions to ask, what kind of documentation needs to be recorded when registering someone in the programme and how to map the participants’ backgrounds and determine what their so-called establishment plan should look like. In addition, the associated administrative support for the clarification of health conditions [44] was made available to the research team.

In order to be in a position to make comparisons, we also inquired about the administrator support document used by case workers during the years prior to the introduction of the new establishment programme in 2018, i.e., the document used by case workers when establishing so-called establishment plans, based on Act 2010: 197 on introduction measures for certain newly arrived immigrants [45]. This was handed to us together with the associated administrative support for performance assessment and a list of activities that could be included in the establishment plans [46,47]. 

## 5. Method

As stated earlier, it is presumed that refugees’ mental health is affected by their level of social participation, as well as their integration or non-integration in the labour market. By adopting a street-level bureaucrat perspective [48] in which we investigated the administrator support document currently used by employment officers in the establishment programme, this study aimed to investigate what these case workers should or can do to address the social participation and mental health of the newly arrived refugees participating in the establishment programme. 

As a first step, a qualitative content analysis was conducted by means of a thematic coding of the content [49] in order to identify the main themes of the administrator support document currently used by case workers in the establishment programme [43]. All three researchers read the administrator support document to get an overview of the material. Two of the researchers then analysed the administrator support document in detail and identified those parts that directly addressed the social participation and mental health of the participating newly arrived refugees, or that indirectly may have some bearing on these aspects. Examples of what could have an impact on social participation and mental health may, for instance, be related to living arrangements, societal demands and cultural beliefs. After the initial identification of parts with bearings on social participation and mental health had been conducted by the first and last authors, the interpretation of the data was continuously discussed and re-evaluated by all of the members of the research team in order to increase credibility. The parts were thereafter categorised into themes. The content of these themes was then compared with the content of the administrator support document used prior to 2018, i.e., that used by Swedish Public Employment Service case workers when creating participants’ establishment plans in accordance with Act 2010: 197. The identified themes and comparisons then served as a basis for a discussion about the degree to which case workers in the Swedish establishment programme took the target group’s specific circumstances and conditions for social participation and mental health into account. Quotes that were especially informative were selected to illustrate the results. Furthermore, attention was paid to the silences relating to the role that social participation and mental health of the participants might have on the programme, or vice versa in the light of the conclusions of previous research.

## 6. Results

In general, documents such as the analysed administrator support document are self-evidently intertextual and interdiscursive, in that they are implementations and interpretations of other texts, such as laws, directives and policy documents [50]. The content of laws and the annual government appropriations determines the Swedish Public Employment Service’s mission and assignments, including what should be achieved through its establishment programme and thus the content of the analysed administrator support document. The most recent years’ government appropriations could be summarised as: the importance of ensuring that all newly arrived refugees who are enrolled in the establishment programme receive high-quality services that allow them to enter the Swedish labour market and to ensure that an equality perspective is adopted in all aspects of the programme [51,52,53]).

The administrator support document used in the current establishment programme guides the case worker through the two phases of the programme. The first phase of mapping is intended to make the participants’ experiences, competencies and conditions for establishment visible. The mapping also includes asking questions about the participant’s health situation and resources, such as motivation, contacts and networks. The second phase, the planning of activities, is meant to increase the participant’s competencies and introduce them to the labour market, mainly through education, training and subsidised employment [43].

The analysis of the administrator support document resulted in the categorisation of four themes which were based on the document’s discursive level and covered the demands stated in the government appropriations directed at the Swedish Employment Service: ‘equal and personalised labour market establishment measures’, ‘individual responsibility and control’, ‘the importance of a gender perspective’, and ‘market needs and matching activities’.

### 6.1. Equal and Personalised Labour Market Establishment Measures

The content of the administrator support document focuses on offering all newly arrived refugees enrolled in the establishment programme high-quality, personalised services. This is showcased, for instance, in paragraphs describing the working methods that case workers are expected to apply. Personalised plans for participants in the programme that take educational and employment background, skills and personal conditions for entering the labour market into consideration are duly stressed [43]).

*“Based on the labour market policy assessment, the participant should be offered activities that are regarded as strengthening the participant’s opportunities for establishment in the labour market and in society. The activities should be planned together with the participant and must reflect the participant’s individual needs.”*

[43] (p. 26)

The support document provides a map of the central discursive categories, such as education, training and labour market introduction programmes when it comes to measures aimed at helping participants to enter the labour market. At the same time, the focus is on aligning the options offered in the programme with the existing labour market initiatives that are already available to other groups of jobseekers listed with the agency [43].

*“Mandatory activities in the Establishment Programme include, as a general rule: Mapping, Swedish studies and social orientation. In addition, there is opportunity to take part of most of the efforts in the Swedish Public Employment Service’s toolbox.”*

[43] (p. 4)

It also acknowledges that other factors may affect the participants’ possibilities of establishing a working life in Sweden, e.g., social conditions relating to family or housing. Examples of such activities include parental support programmes, programmes aimed at guiding participants to better health and study circles. However, these types of activities are not described in detail in the support document and come under the jurisdiction of external organisers, such as the municipality, the county council or independent associations [43].

The content relating to equal and personalised labour market establishment measures in the current administrator support document is similar to that used before the new establishment programme was put in place in 2018 [45]. This document states that: ‘All establishment plans must be based on a labour market policy assessment’ (p. 41) … ‘The activities of the establishment plan must reflect the jobseeker’s individual needs based on the labour market policy assessment’ [45] (p. 41). In addition, it provides a list of topics that must always be included when discussing the content of the establishment plans with the participant, e.g., the jobseeker’s education and professional competence and their performance ability and conditions for participating in activities outlined in the plan [45]. Thus, there are no major changes in the current administrator support document in this respect as a result of the introduction of the establishment programme in 2018.

### 6.2. Individual Responsibility and Control

Another theme discerned in the administrator support document is that of the participants’ own responsibility for their integration. The importance of following up and controlling participants’ progress is clearly stated in the current administrator support document. In particular, it focuses on how to deal with cases where individuals enrolled in the programme are not participating as intended [43].

*“It is you as an employment officer who investigates and makes decisions about revocation of program decision. Before deciding on revocation, the participant must be notified. This means that the participant may take part of the documentation that the decision will be based on and given the opportunity to comment on this. (…) If the participant (…) has submitted comments that make the reasons for the decision on revocation no longer sufficient, the case is dismissed.”*

[43] (p. 40)

In comparison, the previously used support document explains how a participant’s establishment plan might need to be adjusted if the participant has not taken part in the activities as intended. With regard to subsidies, the document states: ‘If information on absence has appeared in the monthly report or from elsewhere, it may be relevant to reduce the establishment allowance’ [45] (p. 27). Hence, the implementation of the current establishment programme instructions on how to follow up on the level of participation and the measures that need to be adopted if an individual is not taking part as intended have become stricter in the sense that they are now more focused on measures of revocation, rather than on an adjustment of activities and the possible reduction in monthly allowances.

### 6.3. The Importance of a Gender Perspective

The importance of adopting an equality perspective, especially between women and men, and for measures to be implemented to increase labour market establishment also among women are recurring aspects of the administrator support document [43] and are reflected in the following way:

*“Statistics show that women are often allowed to take part in, for example, labor market training and work training to a lesser extent than men. Remember to have a gender equality perspective when you discuss activities with the participant.”*

*“If the newly arrived person’s child has not been offered preschool or similar educational care according to the Education Act they may take part in the programme on a part-time basis. (…) The rule has been added from a gender equality perspective and offers families with two guardians equal opportunities to participate in establishment initiatives if they can share the responsibility for their children’s care. It is also possible that the participant is able to find a different solution for the care of their child and thus participate part-time.”*

[43] (p. 16)

As female participants in the establishment programme lack education and/or paid labour experience more often than men, the following statement could also be interpreted as a measure aimed at increasing equality among participants:

*“When there is a lack of work experience, it is even more important to highlight the knowledge and skills that may have been acquired in ways other than through traditional professional work.”*

*“In order to map the participant’s competencies you need to ask questions, partly about the competences gained from previous work, partly about which interests, characteristics and general skills are of particular importance so that the participant can be matched with employment. The starting point of the conversation should be the participant’s abilities.”*

[43] (p. 22)

Even though also the previous administrator support document states that it should be possible to ‘extend an establishment plan due to part-time parental leave to make it easier for parents to combine parenthood with participation in establishment initiatives’ [45] (p. 30), it is clear that the definite demand that employment officers adopt a gender equality perspective is something that came into force with the establishment programme introduced in 2018. 

### 6.4. Market Needs and Matching Activities

The administrator support document used in the establishment programme and the previous establishment plan are both guided by an awareness that the participants are a diverse category in which it is important to discern between individuals with varying levels of experience and education.

Educational measures were also common as part of a participant’s establishment plan before the introduction of the establishment programme in 2018; for instance, the list of possible activities [47] then used includes adult education. However, since the introduction of the establishment programme in 2018, it has become a required measure to adopt in case a participant lacks education for success in the labour market.

*“If you assess that a participant will not be able to be matched against employment during their time in the establishment programme and that this is due to the participant having an incomplete upper secondary education, the programme’s activities should mainly consist of regular adult education in combination with Swedish language studies and social orientation.”*

[43] (p. 25)

Moreover, the currently used administrator support document has a stronger focus on matching participants with professions, e.g., vocational training in collaboration with folk high schools (independent adult education colleges), especially since 2020, when the government gave the Swedish Public Employment Service the task of introducing a so-called intensive training year programme for highly motivated newly arrived refugees with the capacity to acquire the necessary complementary training in a year or less to secure employment.

In general, activities other than those with obvious links to the labour market are less prominent in the present administrator support document compared to the previous one. For instance, possible preparatory activities in the previous establishment plans, such as applying for an ID card, learning to use public transport, association life or study visits, are no longer listed in the current establishment programme.

### 6.5. About Health Aspects

During the mapping phase of the current establishment programme, the case worker is expected to ask participants about their health status using a questionnaire consisting of ten pre-determined questions to explore whether they need medical or other kinds of health support to be able to participate in the programme. However, only one of these questions directly relates to the participant’s mental health; ‘Do you feel anxious/depressed? How and over what?’ [44]. If the case worker thinks that there is a need to map the ability of the participant further, they can seek help from the Swedish Public Employment Service’s specialists or employment agencies with in-depth knowledge of working life rehabilitation. If the participant needs to contact the health care services, the administrator support document urges case workers to refer them to a nearby primary health care clinic and to assess whether there is a need for future collaborations with the health care services. Furthermore, the case workers should adapt their ways of communicating with the participants if needed, for instance, by having several shorter meetings, taking breaks and/or repeating certain information. They can also distribute the information sheet entitled: ‘Do you need support to be able to participate in the Employment Service’s activities?’, which is available in several languages [43].

In comparison, the administrator support document used in the establishment plan prior to the current establishment programme was accompanied by a fourteen-page document called ‘capability assessment’ [46], the aim of which was to guide case workers in the assessment of an individual’s capability of participating in activities in the establishment plan, as well as their capability to function in the labour market. The content is similar to the measures suggested in the current administrator support document but is more detailed when it comes to which adaptations should be applied to enable participants to take part in the establishment plan successfully.

## 7. Discussion

### 7.1. Equal and Personalised Labour Market Establishment Measures

When the current establishment programme replaced the previous establishment plan, the purpose was to focus on accelerating and facilitating refugees’ establishment in the Swedish labour market. Moreover, while previous government initiatives aimed to facilitate newly arrived refugees’ integration into Swedish society as a whole, the current establishment programme is defined as a labour market policy programme [35]. The changes that were implemented also aimed to mainstream the working methods in the establishment programme to better match those of other labour market initiatives and to increase the equality aspect of the programme to ensure that all participants are treated equally [43].

Research has shown that newly arrived refugees constitute a group of job seekers with different characteristics and needs than other groups listed with the Swedish Public Employment Service. Newly arrived refugees encounter additional obstacles when establishing themselves in the labour market in the host country, e.g., due to mental illness [16] or a lack of social capital in the form of social networks [54]. It has also been shown that refugees have lower employment rates, more unstable employment contracts and lower incomes than other groups due to economic, political, social and cultural barriers [32].

Taking these research findings into account and the current establishment programme’s aspirations to adopt equal and personalised labour market establishment measures, it is noteworthy that the specific circumstances under which newly arrived refugees enter the Swedish labour market, in terms of mental health aspects, are so scarcely acknowledged. In the same way, it is notable that few initiatives in the programme are adapted to the specific needs of many newly arrived refugees, and that despite the research available on the role of social participation and its beneficial impact on mental health and labour market integration, the changes that were implemented in 2018 with the establishment programme meant less focus on so-called social activities that are not directly related to educational measures and the labour market. This concern is also shared in an evaluation of the establishment programme by the Swedish Agency for Public Management, which states that there is a risk that individuals who first and foremost need to be established in Swedish society will not be as prioritised as those who are closer to the labour market [38].

### 7.2. Individual Responsibility and Control

In much the same way, aspects of mental health are not really taken into account in relation to participants in the present establishment programme, in that they are expected to manage the process of approaching the labour market themselves and report regularly on their participation and progress in the programme in the same way as other groups of jobseekers listed with the Swedish Public Employment Service [35]. Given the possible language barriers and a lack of understanding about how the different reporting systems function, as well as possible reduced mental health, it may be difficult for some migrants to shoulder responsibility for the establishment process without personal guidance from case workers at the employment agency. The analysed document does not really address how this system of control could affect case workers’ appraisals of the participants’ mental health or their conditions for social participation in other settings, and how these may affect or be affected by a decision on revocation. This is also showcased in the evaluation report by the Swedish Agency for Public Management, where case workers at the agency expressed frustration at not being able to help individuals in the same way as before. On the contrary, the report states that sanctions risked disrupting an individual’s establishment process in that they could lead to anxiety and a reduced confidence in the Swedish Public Employment Service [38]. Nevertheless, it can be argued that the harmonised sanction rules can facilitate participants’ possible future transitions to other labour market policy programmes and function as an educational measure when participants are no longer part of the establishment programme and are expected to approach the labour market and the Swedish authorities with the same level of assistance as the rest of the population. 

### 7.3. The Importance of a Gender Perspective

In the establishment programme, newly arrived refugee men take part in various work-oriented initiatives to a greater extent than their female counterparts. Furthermore, it is more usual for women to take a break in the programme due to parental responsibilities.

One of the salient discursive themes of the government appropriations over the last few years is the equality aspect of the establishment programme and the need for the Swedish Public Employment Service to adopt measures that ensure that no participant is disadvantaged on the grounds of gender [51,52,53]. These demands from the government have influenced how the administrator support document is formulated and the way it requires case workers to adapt and plan the participants’ activities in order for women and men to have equal access to them. However, according to the report by the Swedish Agency for Public Management, case workers at the employment agency assess that men are more matchable to employment than women, even when underlying factors such as age, education and work experience are taken into account [38]. Furthermore, there is still an imminent risk that newly arrived women will not return to the Swedish Public Employment Service after having been away from the programme due to maternity leave [38].

Additionally, the organisation of the current establishment programme and its possibilities to achieve gender equality are affected by other societal and political forces, such as the implementation of the Temporary Act mentioned at the beginning of this article [5,8,9]. According to the Temporary Act, newly arrived refugees have to prove that they can support themselves and their families in order to be granted an extended residence permit, which in many families results in the men taking responsibility for entering the labour market because they are perceived as having a greater chance of success, while the women tend to take care of the children and household [41]. How the establishment programme and the Temporary Act are affected by or affect the mental health of the participants and their families and whether there are disparities between women and men in this respect is not addressed in the analysed material.

### 7.4. Market Needs and Matching Activities

According to a report by the Swedish Public Employment Service [55], research shows that migrants to Sweden find it more difficult to get jobs for which they are qualified than people born in Sweden. They are also more often referred to part-time jobs and temporary employment. The same report states that if those who are highly educated get qualified jobs, there will be less competition for unskilled jobs, which would enable people with lower education levels to stand a better chance of entering the Swedish labour market.

The administrator support document does not define what a successful match entails when it comes to a participant gaining employment after taking part in the establishment programme. For instance, does it mean that a participant is offered employment due to reaching a certain ‘minimum level’, or does it mean that they are employed in the profession for which they are educated? In practical terms, is it considered a success if a participant who used to work as a teacher and who would also like to work as a teacher in Sweden can only find employment as a driver due to having a driving licence?

The idea of matching jobseekers with jobs for which special qualifications are required may have more long-term consequences at an individual and societal level than is acknowledged in the administrator support document. For instance, the long-term effects for those who are highly educated yet end up with jobs requiring less education and training through the establishment programme could be a lack of social participation, loss of a sense of belonging and deteriorating mental health.

One of the cornerstones of the government appropriations that is reflected in the administrator support document is the demand for compulsory education measures. Although the equality aspects have increased compared to previous documents in the sense that the same rules now apply to all newly arrived refugees participating in the establishment programme, and that the programme is more comparable to other employment policy programmes, the education obligation only applies to newly arrived refugees and could therefore be argued to go against the government’s intention to increase equality for all jobseekers. In the Swedish Agency for Public Management’s evaluation report, the interviewed case workers explained that the compulsory education clause sometimes impaired the case workers’ ability to adapt to the participants’ needs. They assessed that newly arrived refugees were guided to initiatives that they were not always ready for or able to cope with, and that other activities were sometimes more appropriate. In this way, the case worker’s professional assessment is subordinate to the rule of the programme [38]. This example of a street-level bureaucrat perspective and how the case workers are torn between the policies and systematisation of the measures they are able to offer on the one hand, and their clients’ individual needs on the other, is not problematised in the current administrator support document. Neither does the support document take the possible effects of compulsory education on the participants’ mental health into account, for instance, if the education requirement is too big a challenge for some individuals.

### 7.5. Mental Health

WHO’s definition of mental health includes perceived self-efficacy, autonomy, competence, intergenerational dependence and recognition of the ability to realise one’s intellectual and emotional potential, which in turn are impacted by individuals’ possibilities to contribute to their communities through labour market participation [12]. However, despite the role that labour market participation has on mental health, and the fact that refugees constitute a particularly vulnerable group of migrants in this respect [1], these aspects are only briefly addressed in the administrator support document.

It is notable that mental health aspects are hardly mentioned in the currently used analysed documents, despite previous research showing that mental health amongst refugees is affected by and affects their levels of social participation and labour market integration in the host country. Additionally, there is no mention of health care specialising in refugee health or other related arenas that could be of significance for the establishment of this group in Swedish society and its labour market. Moreover, the possible negative impact that the Temporary Act and the restricted asylum rules may have on the mental health of participants is not considered. Finally, what happens to those who are not able to continue to take part in the establishment programme due to ill mental health is not clear, as there does not seem to be any alternative programme, such as initiatives focused on integration into society as whole rather than solely into the Swedish labour market.

## 8. Conclusions

The establishment programme and its outcomes have bearing on public health, as it affects the well-being of a societal group by recognising their abilities and aiming to help them to work productively and contribute to their communities [12]. 

The results of this study show that the content of the analysed administrator support document used by case workers reflects the demands stated in the government appropriations addressed to the Swedish Public Employment Service. The document focuses on four discursive themes: ‘equal and personalised labour market establishment measures’, ‘individual responsibility and control’, ‘the importance of a gender perspective’ and ‘market needs and matching activities’. The measures mainly consist of education and job training, as well as measures that are intended to increase equality together with the individual responsibility of the programme participants. Research has shown that newly arrived refugees are a specifically vulnerable group in terms of mental health and social participation. Nevertheless, the analysis of this study shows that the currently used administrator support document pays little attention to these aspects, and to an even lesser extent than the previously used support document, or to the possible effects this may have on the programme and the participants’ integration into the labour market or Swedish society as a whole, as well as the effects this may have on, for instance, the Swedish health care system and other societal institutions.

It is obvious that more research is needed to investigate how the establishment programme takes the participants’ mental health and conditions for social participation into account. Suggested future studies could examine whether and to what extent these factors affect the outcome of the programme and make comparisons with similar programmes in other countries.

## Data Availability

Not applicable.
